# Biomimetic Strategies for Bone Repair and Regeneration

**DOI:** 10.3390/jfb3030688

**Published:** 2012-09-20

**Authors:** Maria G. Raucci, Vincenzo Guarino, Luigi Ambrosio

**Affiliations:** Institute of Composite and Biomedical Materials, National Research Council of Italy, P.le Tecchio 80, Naples 80125, Italy; Email: vguarino@unina.it (V.G.); ambrosio@unina.it (L.A.)

**Keywords:** biomimetic coating, bone substitute, hydroxyapatite, scaffold

## Abstract

The osseointegration rate of implants is related to their composition and surface roughness. Implant roughness favors both bone anchoring and biomechanical stability. Osteoconductive calcium phosphate (Ca-P) coatings promote bone healing and apposition, leading to the rapid biological fixation of implants. It has been clearly shown in many publications that Ca-P coating accelerates bone formation around the implant. This review discusses two main routes for the manufacturing of polymer-based osteoconductive scaffolds for tissue engineering, namely the incorporation of bioceramic particles in the scaffold and the coating of a scaffold with a thin layer of apatite through a biomimetic process.

## 1. Introduction

Each year more than one million patients worldwide need to be treated for skeletal problems which fall within the scope of plastic and reconstructive surgery, orthopedic surgery, and dental implantology. Surgery includes the treatment of bony defects generated by trauma or by the excision of tumors, the reconstruction of congenital skeletal abnormalities, the promotion of fracture healing, the treatment of spinal arthrodesis and the replacement of joints and teeth [1,2,3]. Treatment does not always solve the problem owing to the poor status of local bone and impaired bone healing. Complicated fractures may fail to heal, resulting in delayed union or non-union. The excision of bone tumors and the treatment of congenital syndromes frequently involve the creation of large bony defects, which need to be filled with autogenic or allogeneic bone. Autogenic bone is of limited availability for grafting purposes and its excavation is associated with donor site morbidity. Therefore suitable and biocompatible substitutes for bone grafts have been sought [4,5,6]. 

Bone substitutes can be divided into three classes, namely (1) osteoconductive; (2) directly osteogenic and (3) osteoinductive. Osteoconductive bone substitutes, such as ceramic materials, do not actively stimulate the bone formation process, whereas directly osteogenic and osteoinductive bone substitutes do [7]. 

Osteogenic materials can, for example, be produced by trapping osteogenic cells within a porous scaffold. Osteoinductive materials can then be prepared by loading such porous scaffolds with osteogenic drugs. 

Alloplastic materials are favored for the filling of bone cavities generated traumatically or by the excision of tumors [8]. An ideal candidate is deemed to be one, which maintains the volume of the defect during the initial phase of healing and is then resorbed and replaced by bone. However, the compact filling of a defect with alloplastic material, with the intention of barring its invasion by soft tissue, must be balanced against the reduced potential for osseous regeneration from the parietal and marginal surfaces [9]. Of the many materials which have been tested for their potential to serve as bone substitutes—such as ceramics, glass, and various polymers [10]—only a few are capable of withstanding the forces operative in load-bearing. Bioceramic hydroxyapatite has been widely employed for nearly 20 years. It is relatively cheap, nontoxic, minimally resorbed, of acceptable compressive strength, and attaches well to hard tissues [11,12]. Its most valuable asset is its ability to facilitate bone apposition [13]. Hydroxyapatite is the most important example of a bioactive calcium phosphate ceramics. There is abundant evidence in the literature that sintered hydroxyapatite is well incorporated into living bone and that it does not undergo any significant biodegradation once it has become bonded to it: the latter feature may be disadvantageous as well as advantageous. Although the static mechanical strength of sintered hydroxyapatite is comparable to that of cortical bone, this material is prone to fatigue failure under conditions of high tensile loading, which renders it unsuitable for applications in load-bearing situations. In general, the prerequisite for a material to be bioactive is its ability to form, or to induce, the formation of apatite upon its surface after coming into contact with bone in the body. This intermediate apatite layer serves to bond the implant, comprising bioactive material, to the bone [14]. Therefore, various techniques have been investigated to form an apatite layer on metals such as titanium in an attempt to make them osteoconductive. Some of the techniques for the formation of surface apatite, which are commonly used in the case of metal-based prostheses, can also be applied to polymeric scaffolds in order to mimic the native microenvironment of bone. In this context, there are two main routes for the manufacturing of osteoconductive scaffolds for tissue engineering: (1) Incorporating bioceramic particles in the scaffold through a variety of techniques [15,16]; (2) Coating the scaffold with a thin layer of apatite through biomimetic processes [17,18,19]. These two routes show advantages and disadvantages, and the latter can be used to make the non-bioactive scaffolds readily fabricated to be osteoconductive.

## 2. Bioactive Composite Scaffolds with Embedded Solid Signals

Human bone is essentially a *composite* material, which strictly assembles into a structure comprising an organic phase, an inorganic phase and cells, to form the natural tissue. The extracellular matter embeds the tissue-specific cells in a highly complex matrix, which consists of the other two components, a non-mineralized phase and a mineralized (hydroxyapatite) phase. The former contains natural polymers such as collagens, glycoproteins, proteoglycans and sialoproteins, which play an essential role both in the control of growth and differentiation of cells involved in the bone remodeling. Meanwhile the inorganic phase, based on the mineral hydroxyapatite and comprising 65%–70% of the total matrix, is responsible for the provision of adequate structural support for loads [20]. 

In light of the above, there is considerable ongoing effort to address the design of composite materials, which include ceramics and polymers, to mimic the microstructural features of bone. Hydroxyapatite (HA) and tricalcium phosphates (TCP) have predominated these studies, because they resemble the natural inorganic component of bone and possess osteoconductive properties [21,22,23,24]. However, HA and TCP also exhibit brittle behavior which is a poor match for the mechanical properties of the natural tissue. 

Natural polymers (*i.e.*, collagen, alginate, agarose, chitosan, fibrin and hyaluronic acid or hyaluronan) and/or synthetic polymers are generally considered as interesting materials to support cell ingrowth in most tissues. They enjoy several advantages including versatility and processability, which enables imparting the desired morphology—*i.e.*, porosity accommodating a wide range of pore sizes and shapes and desired mechanical response [25]. Physical-chemical properties of polymer matrices can be easily modified and the mechanical behavior and degradation rate can be suitably tailored by varying the chemical composition. However, these polymers show a bioinert surface that generally lacks bioactive functions for bone formation and, consequently, elicit minimal tissue response. The incorporation of additive chemical functionalities is therefore required in order to improve their chemical bioactivation [20]. Recently, much attention has been paid to the development of polymer/ceramic composite scaffolds, seeking to combine the key features of the individual components in order to obtain functionally active bone substitutes [26,27,28]. Several technological strategies have been successfully invoked to integrate ceramic and polymer phases into porous scaffold systems, including phase inversion/particulate leaching [29] and filament winding technologies [30], rapid prototyping [31,32,33,34], phase separation [35] and emulsion/freeze drying [36]. 

The majority of synthetic matrices show hydrophobic surfaces, which are unfavorable towards basic cell interaction mechanisms (*i.e.*, adhesion, proliferation) compared to hydrophilic surfaces [37,38,39,40]. The inclusion of bioactive solid signals into the polymer matrix may support the formation of a strong bond with the living host bone at the scaffold/implant interface, due to the improved wettability arising from the presence of the apatite particles [41,42]. Consequently, synthetic polymer matrices made of biocompatible polyesters (*i.e.*, polycaprolactone, polylactide acid) have generally demonstrated a tendency to be inert and to promote the formation of encapsulated ﬁbrous tissues. In contrast, the addition of calcium phosphate particles to biodegradable porous matrices offers several improvements which combine to promote bone osteogenesis, as reported in the case of highly porous composite scaffolds made of polycaprolactone (PCL) and stoichiometric HA particles developed through phase inversion and salt leaching techniques [43]. In this case, results highlighted that the presence of HA enhances the scaffold bioactivity and human osteoblast cell response, indicating their role as “bioactive solid signals” in the promotion of surface mineralization and, consequently, cell-material interaction. In particular, the biological studies performed on structures with twofold larger pore size and fully interconnected porosity, characterized respectively by different PCL/HA volume ratio at the same processing conditions, showed that stromal cells from bone marrow (bMSC) were able to adhere and grow on PCL-based scaffolds at any HA content, identifying them as precursors with high replicative potential. Indeed, even though cultured *in vitro* in static conditions, without additional stimulants (e.g., growth factors), MSC adhered during the first four weeks of culture showing a cuboidal appearance on the polymer surface, which is a typical feature of mature osteoblasts. However, in some cases, the presence of HA in PCL scaffolds only slightly affects the biological response and the viability and MSC differentiation appears not to be directly related to the amount of HA in the matrix [44]. Besides osteoconductive enhancement, the relative HA content influences the intrinsic mechanical response of the composite scaffold and degradation properties. 

Several papers have demonstrated the active role of hydroxyapatite filler on the underlying *in vitro* degradation mechanisms by the simultaneous assessment of the influence of scaffold morphology and the physicochemical properties of the porous scaffolds. The addition of HA particles was found to slightly modify the pore morphology, with a small reduction in average pore size. More interestingly, other studies on the scaffold mass losses indicated that the presence of apatite phases embedded in the PCL matrix drastically increased polymer crystallinity. This promoted the formation of more densely packed crystalline phases within these composites. The attendant reduction in the extent of amorphous regions in these materials renders them less susceptible to hydrolytic attacks, which are facilitated by better accessibility of the ester linkage in amorphous domains [45]. In this case, the increase in crystallinity of polymer matrix in HA-loaded scaffolds hinders the degradation of the composites, preferentially deflecting the fluids at the polymer/ceramic interface, which are more susceptible to hydrolytic attack. 

The use of rigid bone-like particles embedded into a polymer matrix evidently improves the mechanical properties of the polymer matrix, strengthening the use of composite scaffolds as a substrate for hard tissue replacement [46,47]. The contribution to mechanical response due to the ceramic phase will be partially reduced by the presence of macro- and microstructured pores, although the latter may be considered a basic requirement to induce the regeneration mechanisms in tissue engineering applications. For this reason, the further integration of biodegradable PLA fibers into the PCL matrix allows improving the mechanical response of the scaffolds, providing spaces required for cellular ingrowth and matrix production. The addition of bioactive apatite-like particles generating needle-like crystals of calcium-deficient hydroxyapatite similar to natural bone apatite also interact with the fiber-reinforced polymer matrix, further enhancing the mechanical response in compression by up to an order of magnitude [48]. 

It should be noted that adverse results have recently been reported from studies of hydroxyapatite-loaded polymer scaffolds where a lack of homogeneity in the distribution of ceramic particles in the polymeric matrix dramatically compromised both the mechanical performance as well as the the bioactive potential of the composite [49]. The polymer matrix degradation, for example, causes a more frequent escape of HA particles in time, with the creation of voids within the polymeric structure [45]. This evidently often affects the mechanical response of the scaffold, influencing the integrity of the scaffolds at longer exposure to *in vitro* culture.

**Figure 1:**
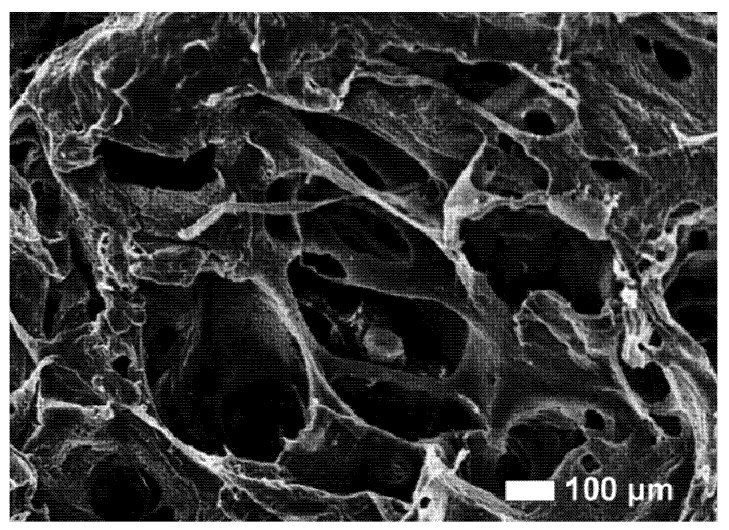
Hydroxyapatite loaded composite material scaffold.

As an alternative strategy, chemically inspired approaches based on the sol-gel transition and colloidal precipitation of calcium phosphates may improve the efficiency of particle dispersion by a direct control of precipitated grain sizes through the interaction between calcium and phosphate precursors under controlled temperature and pH conditions [50]. The sol-gel reaction has been reported to facilitate the introduction of finely dispersed calcium phosphate nanoparticles into a polycaprolactone (PCL) matrix comprising, for example, an improvement in functional properties (*i.e.*, mechanical response, bioactivity) [51,52]. Moreover, presence of HA particles (Figure 1) in the composite material compensates for the acidic release from the polymer with the presence of alkaline calcium phosphate [53]. Whilst it is recognized that a problem with biodegradable polyesters may be acidosis caused by the (chemically unavoidable) release of acidic degradation products, careful *in vivo* and *in vitro* measurements of pH in bone chambers have shown that the pH drop is 0.2 units near the eroding polyesters [54]. The incorporation of bioceramic particles into biodegradable polymers to form bioactive scaffolds overcomes some limits of physically embedded particles through a more efficient physical distribution of the bioactive signal into the scaffold, which improves the bone bonding. In addition, the scaffold interaction at the tissue interfaces may be further improved by the exposure of tailored surface topographies with features in the same scale-range as that seen on the pre-existing bone surface at bone remodeling sites [55]. It has been shown that bone-like apatite coating could form *in vitro* on the composite scaffolds [56,57,58], indicating osteoconductivity of these materials [54,59]. This route of making bioactive bone tissue engineering scaffolds is being explored, with several manufacturing techniques being currently actively investigated.

## 3. Fabrication of Tissue Engineering Scaffolds by Biomimetic Deposition

Currently, tissue engineering scaffolds are typically made from biodegradable polymers such as poly(lactic acid) (PLA), which are non-osteoconductive, and there are various methods to fabricate tissue engineering scaffolds [60], such as by plasma spraying [61], hydrothermal-electrochemical treatment [62], spraying-and-sintering [63] and ion beam assisted deposition [64,65]. The methods used for depositing layers of calcium phosphate upon implant surfaces employ non-physiological, frequently extreme conditions (with temperatures sometimes in the order of several thousand degrees Celsius), which preclude the incorporation of thermolabile signaling substances. Therefore these agents can be deposited superficially only upon preformed coatings, either by adsorption [66,67] by binding to biofunctional proteins [68] or by chemical treatment [69]. The disadvantage of this mode of attachment is that the biologically active molecules are released rapidly upon exposure to a physiological environment [70,71]. Consequently, their osteogenic effects are short ranged and short lived [72].

A few years ago, attempts were made to coat implants with layers of calcium phosphate under more physiological or “biomimetic” conditions of temperature and pH [11,73], primarily to improve their biocompatibility and biodegradability. The mineral layers generated by existing methods, being composed of large, partially molten hydroxyapatite particles, were not only prone to delamination but also degraded in a biological environment [74]. An additional advantage of the biomimetic method is that biologically active molecules, such as osteogenic agents, can be co-precipitated with the inorganic components. Consequently, the proteins are properly incorporated into the crystal latticeworks and not merely deposited upon their surfaces. In forming an integral part of the calcium phosphate coatings, the protein molecules are liberated not in a single burst—as they are when superficially adsorbed—but gradually, which bodes well for an enduring osteogenic effect at the implantation site. The biomimetic coating technique involves the nucleation and growth of bone-like crystals upon a pretreated substrate by immersing this in a supersaturated solution of calcium phosphate under physiological conditions of temperature (37 °C) and pH (7.4). The method, originally developed by Kokubo in 1990 [75], has undergone improvement and refinement by several groups of investigators [76,77,78,79]. It is simple to perform, is cost-effective and may be applied even to heat-sensitive, non-conductive and porous materials of large dimensions and with complex surface geometries.

Until recently, the majority of the biomimetic routes proposed for coating of Ca-P layers on the surface of biomaterials have been limited to static conditions [80,81,82]. However, *in vivo*, the mineralization of bone tissue occurs in the presence of body fluids, which continuously circulate in the body [83]. Accordingly, an *in vitro* biomimetic approach including dynamic studies is of great significance, as it comes closer to the *in vivo* scenario, where the flow of human body fluids may have an effect on the formation of bone apatite [84]. Some authors [85,86] have studied the mineralization of apatite layers under dynamic conditions, though these studies were only intended to better assess the bioactive behavior of silica-based bioceramics. These materials are highly reactive and cause a local decrease of Ca^2+^ and PO_4_^3^^−^ in the surrounding solution during apatite formation in static conditions, which will compromise the progress of the mineralization process. When considering 3D porous architectures that are not intrinsically bioactive, dynamic mineralization environments can also be suitable to promote a homogeneous formation of the Ca-P layer on their interior. Indeed, dynamic conditions can also accelerate the process for apatite formation in a pre-established apatite layer while maintaining the composition, crystallinity and chemical structure of the apatites. A small number of studies [85,86] have addressed the induction and growth of an apatite layer on the surface of bioactive materials in dynamic simulated body fluid (SBF): Table 1 sets out the ionic composition of SBF and human blood plasma, which are very similar [87,88]. To the authors’ knowledge, this kind of approach has been only proposed for accessing the SBF *in vitro* mineralization of materials with intrinsic bioactivity, such as silica based ceramics [89] or their composites [85]. Besides better mimicking bone mineralization, the rationale for these studies was also intended to avoid the local build-up of released silicic acid and ions from the silica-containing materials to the SBF solutions, which influence the formation of the apatite layers.

**Table 1:** The ionic concentration of human blood plasma and simulated body fluid (SBF).

Ion concentration (mM)	Na^+^	K^+^	Ca^2+^	Mg^2+^	HCO_3_^−^	Cl^−^	HPO_4_^−^	SO_4_^2^^−^
**Blood plasma**	142	5	2.5	1.5	27	103	1	0.5
**SBF**	142	5	2.5	1.5	4.2	148	1	0.5

### 3.1. Biomimetic Coating on Metals and Glass-Ceramics

According to the literature and the authors’ experience, a coating, which is successful in enhancing the osteointegration of a metal implant, must be thick and sufficiently crystallized to accommodate the bone healing process. Some authors have reported [90] a treatment comprising Ti_6_AL_4_V with a modified SBF solution containing crystal growth inhibitors (Mg^2+^ and HCO_3_^−^). By immersing cleaned Ti_6_Al_4_V directly into the modified-SBF solution, a loose and nonuniform layer was obtained, indicating the importance of amorphous precoating obtained with a preliminary treatment in SBF solution: This is why a general biomimetic coating consists of two steps. Immersion of the implants in a SBF solution is necessary to seed the metal surface with calcium phosphate nuclei. During this nucleation process, calcium phosphate seeds are precipitated in the solution and on the metal surface. Some of these nuclei can dissolve in the solution and some can expand in size. Homogeneous nucleation (precipitation) occurs spontaneously in the solution and can proceed if other seeds form in the meantime.

Heterogeneous nucleation, on the other hand, takes place on the metal surface: Both homogenous and heterogeneous nucleation are in competition during the process in the modified SBF solution. However, nuclei are energetically more stable on the seeded metal surface than in the solution. It is, therefore, essential to provide the metal surface with a thin and uniform primer calcium phosphate layer for subsequent growth of the final coating. The kinetics of the process in the SBF solution was reported in detail by Barrére *et al.* [91]. 

After reaching their critical size, seeds can start growing into crystals. The nucleation and growth kinetics of the crystal depend on the temperature, pH, composition, and saturation of the solution. Calcium and phosphate ions are responsible for the formation of the calcium phosphate layer on the metal surface, while magnesium and carbonate ions favor heterogeneous nucleation rather than crystal growth. A crystalline apatite phase, resulting from lower amounts of these so-called crystal growth inhibitors, is formed and a drop in the pH is observed at the start of precipitation by immersion in the modified SBF solution [92,93,94,95,96,97,98,99]. A smaller amount of HCO_3_^−^ ions, compared to the SBF solution, decreases the buffering capacity of the CO_2_/HCO_3_^-^ couple and so variations in the pH can be observed.

With a lower amount of Mg^2+^ in the modified SBF solution, the calcium phosphate precipitation is accelerated, and the growing coat becomes more crystallized. Between the end point of crystal growth and the end of the process, equilibrium is achieved between the amount of calcium and the amount of phosphate in the coating and in the solution. However, the coating may dissolve and reprecipitate onto the surface, resulting in a more homogeneous and dense coating at the end of the process.

More recently, these biomimetic strategies have been adapted to new bioactive glasses and glass-ceramics which represent a class of materials largely used in bone regeneration [100] whose surface reactivity in contact with biological fluids has been widely studied. The growing interest in these systems is based on their ability to induce *in vitro*, by immersion in a simulated body fluid (SBF), the formation of a semicrystalline hydroxycarbonatoapatite (HCA) rich layer. This behavior is considered an indication of their *in vivo* bioactivity (natural bonding to living tissues) through a mechanism that involves the formation of a “bio-like” layer on the material surface. As reported in the literature [101], the bioactivity mechanism starts with rapid ion exchange between the alkaline ions from the glass surface and the hydrogen ions from solution, followed by the formation of silanols, which then undergo polycondensation to develop a silica gel layer. This layer promotes the adsorption of Ca^2+^ and PO_4_^3^^−^ ions from solution. These ions subsequently react, forming the HCA layer. This mechanism, frequently reported in the literature, has been observed in MgO-containing glasses as well as in MgO-free glasses. Some authors reported a decreased ability of apatite formation in glass ceramic with a higher MgO content. It is known that SiO_2_ glass is bioactive, *i.e.*, it is able to bond to living bone [102]. FTIR analyses reveal the formation of a hydroxyapatite layer by the appearance of the 1116 and 1035 cm^−1^ bands, usually assigned to P-O stretching, and of the 580 cm^−1^ band usually assigned to the P-O bending mode [103]. The splitting, after only 7 days of soaking, of the 580 cm^−1^ band into two others at 610 and at 570 cm^−1^ can be attributed to formation of crystalline hydroxyapatite [104]. Finally, the band at 800 cm^−1^ can be assigned to the Si-O-Si band vibration between two adjacent tetrahedra characteristic of silica gel [105]. This supports the hypothesis that a surface layer of silica gel forms as supposed in the mechanism proposed in the literature for hydroxyapatite deposition [106,107]. 

**Figure 2:**
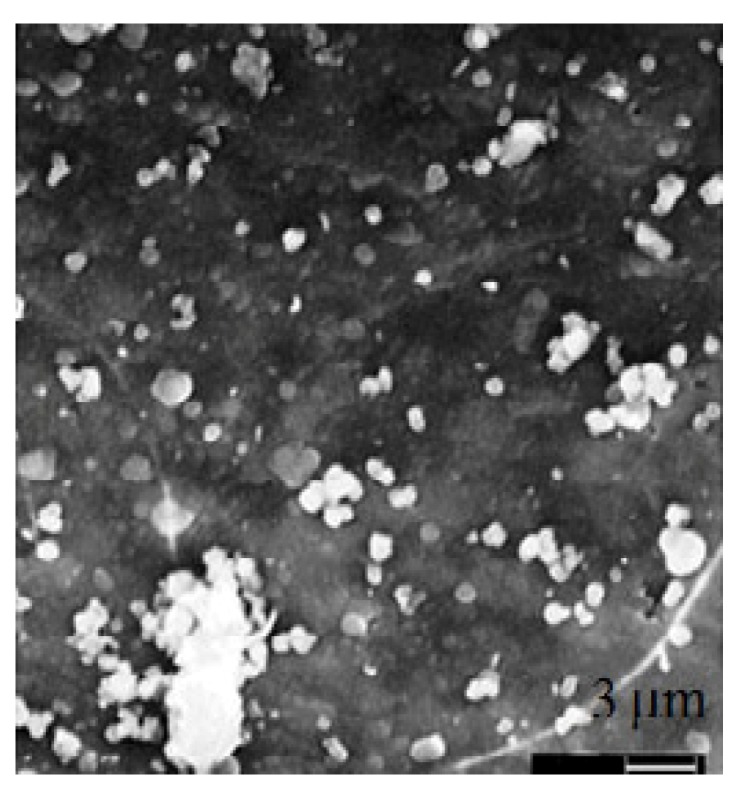
Hydroxyapatite nuclei deposition on glass-ceramic after 7 days of incubation time.

Catauro *et al* have demostrated that silica gel, obtained by sol-gel method, such as Na_2_O-CaO-2SiO_2_ gel and Na_2_O-CaO-2SiO_2 _gel containing 0.50 wt % Ag_2_O show a bioactive hydroxyapatite layer after 14 days of SBF treatment. FTIR measurements and SEM micrographs (Figure 2) have indicated the formation of a hydroxyapatite layer on the surface of samples soaked in a simulated body fluid for different periods [108]. In contrast, calcium phosphate ceramics containing zinc were recently developed to exploit the advantageous effect of released zinc on bone formation. Zinc acts as an essential trace element that has stimulatory effects on bone formation *in vitro* and *in vivo*. The addition of zinc to a bioactive glass-ceramic may serve to control the reaction between the glass-ceramic and the surrounding body fluid, and also the released zinc ion from the glass-ceramic may enhance bone regeneration. It has also been reported that a glass-ceramic containing zinc oxide such as the system ZnO-CaO-SiO_2_-P_2_O_5_-CaF_2_ facilitates the production of superficial apatite [109].

### 3.2. Biomimetic Coating on Polymers

Various coating techniques for deposition of apatite on the surfaces of polymeric materials have been developed over the past two decades [110,111,112,113,114]. 

**Figure 3:**
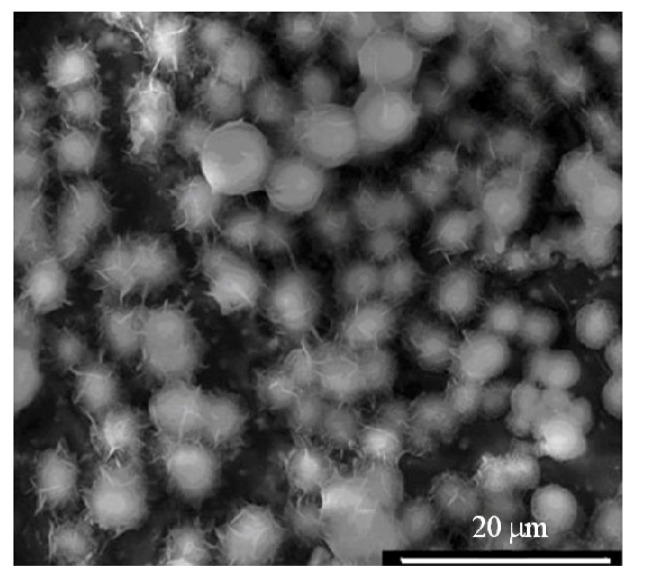
Biomimetic coating on polycaprolactone (PCL) scaffold after 14 days of incubation time.

For example, Taguchi *et al.* proposed an alternate soaking process [111], in which a polymer substrate is alternately and recurrently soaked in calcium ion and phosphate ion solutions. Moreover, it is possible to modify the polymer surface with functional groups effective in inducing apatite nucleation followed by immersion in SBF. Si-OH [114], Ti-OH [115] and carboxyl or carboxylate [116] groups have been used as the functional groups. The mechanism of apatite formation on the surface-modiﬁed polymer is believed to involve functional groups on the polymer surface inducing apatite nucleation in SBF. The induction period required for the apatite nucleation is dependent on the kind [117], number [114] and arrangement of the functional groups. Once the apatite nuclei are formed, they grow spontaneously into a dense and uniform layer of bone-like apatite (Figure 3) by consuming calcium and phosphate ions from the SBF, since SBF is supersaturated with respect to apatite [118]. The period required for inducing apatite nucleation is therefore a critical factor in the apatite-forming ability of the polymer in SBF. 

Although various surface modiﬁcation techniques have been proposed to coat a polymer surface with bone-like apatite [119], they are all aimed at induction of apatite nucleation on the polymer surface in SBF. The surface modiﬁcation provides a polymer surface with nuclei or precursors of apatite (*i.e.*, bioglasses), prior to the immersion in SBF, and this causes shortening of the period required for apatite deposition from SBF. Biodegradable polymers such as polycaprolactone (PCL) have been used for bone tissue engineering (TE) scaffolds; the material surface must be modified to support the cell attachment, proliferation and differentiation (Figure 3). It has been reported that nanocrystalline apatite can facilitate the osteoinductivity and osteoconductivity of the polymer scaffolds [120]. NaOH surface treatment of PCL is a prerequisite for inducing apatite formation. This is mainly due to the PCL hydrolysis, resulting in chain scission of the polyester chains in PCL and formation of carboxylic acid ligands on the surface. FTIR spectra show a new peak around 1560 cm^−1^, which is representative of –COOH. This peak grows as the NaOH concentration increases. Carboxyl ligands have been reported to induce apatite formation through strong binding to positively charged Ca^2+^ ions, forming nuclei, which undergo subsequent growth to apatite. In this case, FTIR data gradually change with immersion of PCL in SBF, the presence of P-O peaks around 1000 cm^−1^ being detectable after 14 days. 

For highly degradable polymers such as hyaluronan derivates (e.g*.*, Hyaff-11^®^), a novel treatment, which combines the preliminary use of bioglasses with supersaturated SBF solutions at different salt concentrations, has been optimized to overcome the applicability limitations of traditional treatments. 

To produce the apatite coatings, scaffolds of hyaluronic acid (HA)-based biodegradable polymer sponge (Hyaff-11^®^) were submitted to a bio-inspired procedure, namely biomimetic treatment, previously described by Tanahashi and co-workers [121]. Manferdini *et al.* [122] have used a treatment that combines the preliminary treatment of a bioglass with a supersaturated SBF solution (5xSBF_1_) to stimulate the nuclei formation with subsequent exposure to a fresh chemically modified solution (5xSBF_2_), in order to promote the growth of apatite nuclei, once formed. The biomimetic treatment consists of two steps in a pH-controlled environment. During the first step, HA-based scaffolds configured as sponges were soaked in 5xSBF_1_ at pH = 6.5, in the presence of pulverized bioglasses of a fragment size of 150–300 µm. The 5xSBFs solution volumes have been calculated with respect to the total scaffold material surface by using a surface exposed to SBF volume ratio equal to 10 mm^2^/mL, as reported in the literature [123]. The solution temperature was fixed at 37 °C during the treatment. After sequential immersion in 5xSBF_1_ (24 h) and in 5xSBF_2_ (48 h), all scaffolds were gently rinsed with distilled water to remove excess ions and then dried overnight under a lamina flow hood. Manferdini *et al*, by accelerating SBF treatments, have demonstrated that this biomimetic HA-based scaffold favors faster induction of the mineralization process, suggesting possible clinical utility as a good cell-carrier or as an alternative bone graft substitute for healing bone defects. The ability of this scaffold to mineralize is exciting and warrants further studies to identify whether this combination (HA/apatite crystal) can also maximize *in vitro* h-MSCs clonogenicity and self-renewal [122].

## 4. Conclusions

This review reports several biomimetic approaches for bone repair and regeneration. Two main routes have been introduced to manufacture polymer-based osteoconductive scaffolds for tissue engineering, namely the integration of bioceramic nanoparticles in the scaffold and the coating of a scaffold with a thin layer of apatite through a biomimetic process. Biomimetic treatments in combination with bioactive bulk inclusions may assure a uniform bioactivation of different substrates by promoting an efficient nucleation and growth of bone-like crystals for bone regeneration. 
